# Peers United in Leadership & Skills Enhancement: A near-peer mentoring program for medical students

**DOI:** 10.36834/cmej.69920

**Published:** 2020-12-07

**Authors:** Adam Neufeld, Zachary Huschi, Amanda Ames, Krista Trinder, Greg Malin, Meredith McKague

**Affiliations:** 1 College of Medicine, University of Saskatchewan, Saskatchewan, Canada; 2 Department of Undergraduate Medical Education, University of Saskatchewan, Saskatchewan, Canada; 3 Department of Academic Family Medicine, University of Saskatchewan, Saskatchewan, Canada

## Implication Statement

We created a near-peer mentoring program in pre-clerkship, which gave medical students the opportunity to work together, teach others, and practice their clinical skills. It uniquely connects first year “learner-mentees” and second year “instructor-mentors” in semi-structured learning environments, from October to April. Beyond supporting intrinsic motivation, skills development, and collaboration, students gained experience in teaching, an important skill for physicians.

## Énoncé des implications de la recherche

Nous avons créé un programme de mentorat par les pairs au préexternat, qui a donné aux étudiants en médecine l’occasion de travailler ensemble, d’enseigner aux autres et d’améliorer leurs habiletés cliniques. Ce programme met en contact de façon unique les « apprenants-mentorés » de première année et les « instructeurs-mentors » de deuxième année et les placent dans des environnements d’apprentissage semi-structurés tout au long de l’année universitaire. En plus de favoriser la motivation intrinsèque, le développement des habiletés et la collaboration , ce programme a permis aux étudiants de se pratiquer à enseigner , une importante habileté pour les médecins.

## Introduction

Medical educators are increasingly recognizing that mentoring is highly valuable in early medical careers.^1^^,^^2^ Most Canadian medical schools offer mentorship programs to facilitate student relationships and overall development. Some medical schools have also adopted near-peer mentoring programs (where one or more advanced students, by at least one year, teach one or more junior students in the same educational program). This approach has been shown to benefit medical learners and mentors in a range of different ways, including the provision of non-threatening learning environments, more choices and freedoms for self-directed learning, less cognitive distance between learners and instructors, and smaller groups which promote connectedness.^1^^,^^2^^,^^3^^,^^4^

Previous mentorship programs have not addressed clinical skills development or teaching. In addition, the majority of mentoring programs focus on mentee development, and less on the mentor.^3^ Therefore, we created a semi-structured near-peer mentoring program grounded in Self-Determination Theory (SDT) to promote learner engagement, academic performance, and psychological well-being.^5^^,^^6^ While near-peer mentoring programs in medical education are prominent outside of Canada,^7^^,^^8^ we believe this is the first near-peer mentoring program in a Canadian medical school, and the first to measure autonomy-support in this context.

## Innovation

We developed a near-peer mentoring program, “Peers United in Leadership & Skills Enhancement” (PULSE) to provide pre-clerkship students with unique opportunities to collaborate and develop their clinical and teaching skills in semi-structured clinical settings. PULSE matches revolving groups of two second-year “instructor-mentors” with two or more first-year “learner-mentees” weekly from October-April, with exceptions for holidays and exams. PULSE operates through a Student Interest Group, which appoints official student coordinators each year (based on application and student vote) and is overseen by faculty members. Each week, 12-18 students could sign up online for sessions that addressed various skills, such as history taking and physical exams (e.g. cardiac, respiratory, musculoskeletal, neurologic, etc.). Learner-mentees and instructor-mentors could enroll as many times as they liked and for whichever skill area they chose. To accommodate multiple learner-mentee groups and promote teaching practice, three separate 1-hour skills sessions were led consecutively by the same instructor-mentors. To promote autonomy, instructor-mentors received only general instructions prior to mentoring, without any specific pre-requisite training. Skills session topics coincided with students’ curriculum and progressed from basic history taking and physical exams, to procedural (e.g. otoscope) and interpretative skills (e.g. chest x-ray), to problem solving (e.g. formulating differentials, investigations, and management plans) and presenting cases. Eventually, skills were combined to mimic Objective Structured Clinical Examinations (OSCEs), which occur twice per year (as a mid-year and an end-of-year exam).

We present some of the learner-mentees’ feedback, along with preliminary data on their responses to an adapted 6-item Learning Climate Questionnaire (LCQ, see appendix),^9^ which measures perceived autonomy-support. According to SDT, autonomy-support facilitates basic psychological need satisfaction—competence (to feel effective and capable of mastery), autonomy (to feel in control of behaviours and goals), and relatedness (to experience a sense of connection with others)—which predicts quality motivation, learning, and well-being.^10^

This project was approved by the University of Saskatchewan Research Ethics Board and students provided written informed consent.

## Outcomes

Between April-June 2018, we held 16 PULSE sessions and distributed several previously validated questionnaires and a general feedback form electronically to PULSE participants. Seventeen of 100 (17%) 2^nd^ year medical students (“instructor-mentors”) and 38/100 (38%) 1st year medical students (“learner-mentees”) participated in PULSE Learner-mentees attended an average of 2-3 sessions (M = 2.8, SD = 0.5), see Table 1. Twenty six learner-mentees (68.4%) completed the survey, containing the LCQ and general feedback form. PULSE was rated as highly autonomy-supportive (M = 6.4, Mdn = 6.7, Min = 4.8, Max = 7.0, SD = 4.0). Participants also reported that PULSE helped reduce performance anxiety around OSCE’s and said, “it not only provided a safe and relaxing learning environment, but comradery and professional relationships as well”… ”the second years were incredibly helpful and had a different perspective than instructors, which made it easier to ask questions”…and “supportive feedback and non-judgmental environment.” Not all students were aware that sessions would progress in difficulty, which negatively impacted repeated attendance.

**Table 1:** Frequency of PULSE sessions attended by learner-mentees

Number of sessions	Number of students	Percentage frequency (%)
1	12	31.6
2	10	26.3
3	7	18.4
4	3	7.9
5	2	5.3
6	1	2.6
7+	3	7.9
M (SD) = 2.8 (0.5)	*n* = 38	100%

M = mean; SD = standard deviation; *n* = sample size

## Next steps

We provide an approach to near-peer mentorship in pre-clerkship that builds on traditional learning goals and supports mentee and mentor development, collaboration, and self-determination. We plan to be clearer to mentees about the progressive nature of the skills sessions, which we believe may support repeat attendance. We intend to explore its effectiveness in more detail, including PULSE’s impact on learner-mentees’ and instructor-mentors’ perceived competence in their clinical and teaching skills, respectively.

## Appendix A

General Learning Climate Questionnaire (LCQ) – adapted for PULSE

This questionnaire contains items that are related to your experience with your instructor in this class. Instructors have different styles in dealing with students, and we would like to know more about how you have felt about your encounters with your instructor. Your responses are confidential. Please be honest and candid.

Use the scale:

**Table:**

1	2	3	4	5	6	7
Strongly disagree			Neutral			Strongly agree

1. I feel that my PULSE instructor(s) provide me choices and options.
2. In general, I feel understood by my PULSE instructors.
3. In general, my PULSE instructors conveyed confidence in my ability to do well in the sessions.
4. In general, my PULSE instructors encouraged me to ask questions.
5. In general, my PULSE instructors listen to how I would like to do things.
6. In general, my PULSE instructors try to understand how I see things before suggesting a new way to do things.

The LCQ has a long version (15-items) and a short version (6 items). The questionnaire is typically used with respect to specific learning settings, such as a particular class, at the college or graduate school level. Thus, the questions are sometimes adapted slightly, at least in the instructions, so the wording pertains to the particular situation being studied—an organic chemistry class, for example. In these cases, the questions pertain to the autonomy support of an individual instructor, preceptor, or professor. If, however, it is being used to assess a general learning climate in which each student has several instructors, the questions are stated with respect to the autonomy support of the faculty members in general.

The 6-item version consists only of items # 1, 2, 4, 7, 10, and 14, from the 15-item version.

**Scoring:** Scores on both the 15-item version and the 6-item version are calculated by averaging the individual item scores. Higher average scores represent a higher level of perceived autonomy support. Information taken from www.selfdeterminationtheory.org website.
